# Synergistic cerium oxide nanozymes: targeting DNA damage and alleviating tumor hypoxia for improved NSCLC radiotherapy efficiency

**DOI:** 10.1186/s12951-023-02196-4

**Published:** 2024-01-10

**Authors:** Jie Liu, Chengxiang Liu, Jinghua Tang, Qiao Chen, Yan Yu, Yan Dong, Jie Hao, Wei Wu

**Affiliations:** 1 Department of Cardiothoracic Surgery, Southwest Hospital, Army Medical University, 30 Gaotanyan Main St, Chongqing, 400038 China; 2 Department of Oncology, Southwest Hospital, Army Medical University, 30 Gaotanyan Main St, Chongqing, 400038 China

**Keywords:** Radiotherapy, NSCLC, Quercetin, ZIF-8, CeO_2_

## Abstract

Radiotherapy (RT) is one of the important treatment modalities for non-small cell lung cancer (NSCLC). However, the maximum radiation dose that NSCLC patient can receive varies little. Therefore, the exploitation of novel RT sensitization approaches is a critical need for the clinical treatment. RT resistance in NSCLC is linked to tumor microenvironment (TME) hypoxia, cell cycle arrest and associated genetic alterations. Here, we designed a novel method for targeted delivery of quercetin (QT) and CeO_2_ to enhance RT sensitivity. We loaded QT into CeO_2_@ZIF-8-HA nanoparticles to prevent its degradation in the circulatory system and successfully delivered QT and CeO_2_ targeted to NSCLC tumors. Under the protection and targeted delivery of Zeolitic Imidazolate Framework-8 (ZIF-8), the nanocomplexes exhibited excellent catalytic mimetic activity in decomposing H_2_O_2_ into O_2_, thus significantly reversing the hypoxia of TME, while the radiosensitizer QT caused DNA damage directly after RT. In a subcutaneous tumor model, CeO_2_@ZIF-8-HA overcame radiation resistance and enhanced therapeutic efficacy. This multiple sensitization strategy combining delivery of QT and CeO_2_@ZIF-8-HA nanozymes opens a promising approach for RT of NSCLC.

## Introduction

Radiation therapy (RT) is currently an irreplaceable treatment strategy for the clinical treatment of many tumors, and it is mostly applied in combination with chemotherapy and surgery. RT, as one of the important adjuvant treatments, has made significant progress in the treatment of non-small cell lung cancer (NSCLC). However, the severe radiation damage caused by RT to adjacent healthy tissues cannot be ignored and, as a result, the maximum acceptable radiation dose to NSCLC patients has barely improved [1]. Not only that, but as the number of RT sessions increases, RT often leads to radiation resistance, which results in a decrease in treatment efficiency [2, 3].

To overcome resistance to RT, many radiosensitizers have been developed and are in clinical use, for example, sodium glycididazole [4]. But these RT sensitizers still have physical toxicity and serious side effects during the treatment of radiation [5]. Therefore, an ideal radiosensitizer is urgently needed to be developed with the following properties: (i) good biocompatibility and biosafety, (ii) outstanding tumor targeting ability, and (iii) excellent RT sensitization efficiency.

Quercetin (QT), a natural flavonoid, has been extensively studied for its anticancer effects in cell signaling, pro-apoptosis, anti-proliferation, and anti-angiogenesis. QT can increase the expression of TSP-1, inhibit angiogenesis, and slow down the growth of cancer cells [6]. Previous studies have shown that QT can interfere with the DNA repair process of tumor cells during RT. Furthermore, both in vitro and in vivo studies have demonstrated that QT can significantly enhance the radiosensitivity of tumors by inhibiting the ATM-mediated pathway [7]. In recent years, the application of QT as a radiosensitizer has been increasingly investigated [8]. Some studies have confirmed the radiosensitizing effect of QT in solid tumors such as breast cancer and prostate cancer [9, 10]. As a highly efficient and low toxic radiation sensitizer, QT is currently a promising direction for radiation sensitization research. QT has poor chemical stability and is prone to oxidation and combustion in neutral and alkaline conditions, which limits its application [11]. Currently, an increasing number of drug delivery systems are being developed to enhance the stability of QT. Melike et al. used poly (lactic-co-glycolic acid) nanoparticles to load QT, which enhanced its cytotoxicity and antioxidant activity against C6 glioma cells [12]. Therefore, using nano-scale delivery carriers to deliver QT to the radiation therapy area is a suitable approach.

A hypoxic tumor microenvironment (TME) is a typical feature of solid tumors and plays a key role in radiation resistance. Reducing tumor hypoxia can effectively reduce tumor resistance to RT [13]. Nanozymes, a nanomaterial with enzyme-like properties, have attracted significant attention due to their distinct advantages over natural enzymes, including high stability in harsh environments, ease of synthesis, and tunability in catalytic activity. Various nanozymes, such as cerium oxide (CeO_2_) [14], manganese (Mn) [15] and Mn/Ce oxide nanoparticles (16), have now been fabricated to achieve effective treatment of hypoxia in the TME. Different nanosystems have been synthesized to modulate intracellular reactive oxygen species (ROS) for therapeutic use. Based on Indocyanine green (ICG), an ROS trigger and photosensitizer, Yi et al. designed a nanosystem combined photodynamic therapy and ROS-responsive chemotherapy [16]. Zhao et al. developed a mitochondria-targeting and protein sulfenic acid (PSA)-reactive gold nanoparticles (dAuNP-TPP) to improve CT imaging and radiotherapeutic efficacy of tumors [17]. However, there are still many obstacles that prevent the further application of nanozymes, such as poor delivery efficiency and elimination half-life. To improve the biocompatibility and bioavailability of nanozymes, nanoscale delivery carriers are ideal candidates. Among them, metal organic frameworks (MOFs) have received a lot of attention and different therapeutic cargoes can be loaded into nanocarriers to exert synergistic effects on tumor therapy [18]. As a metal-organic framework nanoparticle, ZIF-8 is formed by the connection of zinc ions with N atoms in 2-methylimidazole. ZIF-8 nanoparticles can achieve targeted tumor accumulation of cargoes by enhanced permeability and retention (EPR) effect and promote cellular uptake of cargoes without degradation [19]. ZIF-8 promotes controlled release of cargoes in acidic TME by disrupting the coordination interaction between zinc^2+^ and imidazole. Moreover, its excellent biodegradability and low cytotoxicity make it an appealing nanocarrier [20].

Based on this, we have proposed a multifunctional radiosensitizer (C/Q@ZIF-8-HA) loaded with both CeO_2_ and QT to simultaneously increase the DNA damage on RT and alleviate tumor hypoxia. C/Q@ZIF-8-HA exhibits strong peroxidase activity in physiological pH or weakly acidic TME. By decomposing H_2_O_2_ into O_2_, C/Q@ZIF-8-HA could significantly alleviate tumor hypoxia and improve the sensitivity of tumors to RT. More than that, high Z element Ce^2+^ can promote intracellular radiation energy deposition and aggravate DNA damage. As an excellent radiosensitizer, QT can effectively improve the sensitivity and tumor killing power of RT. Passive targeting has limited internalization of tumor cells due to the EPR effect associated with defective tumor vasculature and leaky structures [21]. We coated hyaluronic acid (HA) on the surface of ZIF-8 with a view to achieving good active tumor targeting ability. These findings suggest that this novel multi-functional nanozyme provides a valuable approach for enhancing the sensitivity of NSCLC to radiotherapy.

## Materials and methods

### Ethics statement

Animal experiments were performed with the approval of the Animal Ethics Committee of the Army Medical University. Female BALB/c nude mice (20 g) were provided by the Department of Laboratory Animals of the Army Medical University and maintained at circadian rhythm (12 h), relative humidity (50%) and ambient temperature (25 °C). All operations were performed following relevant ethical regulations.

### Cell lines and materials

A549 and NHBE cells were provided by the Department of Oncology, Southwest Hospital, Army Medical University. QT, zinc nitrate and dimethylimidazole was prepared by RuiXi Materials Tech Co. (Xian, China). Fluorescein isothiocyanate (FITC) were obtained from Aladdin Reagent Inc. (Shanghai, China). CCK-8 assay kit were obtained from beyotime Inc. (Shanghai, China). Hypoxia Inducible Factor 1 Alpha (HIF-1α) ELISA kit was obtained from Jianglai Bio Co. (Shanghai, China). Annexin V-FITC/PI Apoptosis Detection Kit was obtained from Vazyme Bio Co. (Nanjing, China).

### Preparation of CeO_2_-ZIF-8-HA and C/Q@ZIF-8-HA

To synthesize CeO_2_-ZIF-8-HA: Dissolve zinc nitrate, dimethylimidazole and QT separately. Add CeO_2_ and QT to the dimethylimidazole solution first and mix well with it, then add zinc nitrate solution to the mixed solution slowly drop by drop. The solution was stirred at room temperature for about half an hour, and the precipitate was washed by centrifugation to obtain C/Q@ZIF-8. The micelle surface was then modified with HA by EDC/NHS cross-linking reaction to obtain the C/Q@ZIF-8-HA with CD44 activity targeting.

To synthesize CeO_2_@ZIF-8-HA: Briefly, dissolve zinc nitrate and dimethylimidazole separately. Add CeO_2_ to the dimethylimidazole solution and mix it well, then add zinc nitrate solution to the mixed solution slowly drop by drop. The solution was stirred at room temperature and reacted for about half an hour, and the precipitate was washed by centrifugation to get CeO_2_@ZIF-8. The micelle surface was then modified with HA by EDC/NHS cross-linking reaction to obtain the CeO_2_@ZIF-8-HA with CD44 activity targeting.

### Characterization methods

A suite of characterization methods was employed. Transmission electron microscopy (JEM-2100, JEOL) was used to observe the morphology of the C/Q@ZIF-8-HA and confirm that CeO_2_ was successfully loaded into the ZIF-8. Fourier transform infrared (FT-IR) spectra were recorded using a Nicolet iS50 FTIR spectrometer (Thermo Scientific). Dynamic light scattering (DLS) (Malvern Zetasizer Nano-ZS90) was used to observe the nanoparticle size and zeta potential of the nanozymes.

### The catalytic activity measurement of C/Q@ZIF-8-HA

The Amplex™ Red Hydrogen Peroxide/Peroxidase Assay Kit was used to evaluate the catalase-mimicking properties. 200 µL of 2 mg/mL nanoparticles (ZIF-8-HA, CeO_2_, or C/Q@ZIF-8-HA) and 1 mL of a 20 µM hydrogen peroxide solution were added to each well of a 24-well plate. After 2 h of shaking, 50 µL of the samples from each well were transferred to a 96-well plate for the next step. A working solution (50 µL) containing 100 µM Amplex Red reagent and 0.2 U/mL horseradish peroxidase (HRP) was added to each well and incubated for 30 min at room temperature. The fluorescence intensity was measured using a microplate reader at excitation and emission wavelengths of 545 and 590 nm, respectively.

### QT release profile

20 mg of C/Q@ZIF-8-HA nanoparticles, weighed accurately, was evenly dispersed in phosphate buffer solutions with pH 5.5, pH 6.8, and pH 7.2. The solutions were sealed and stirred in the dark. Then, at specific time points (0, 1, 2, 4, 6, 12, 24, 36, 48, 72, 96 h), the samples were centrifuged, and the supernatants were collected for UV analysis. The absorbance of the supernatant was measured at 374 nm wavelength, and the amount of QT was calculated using a calibration curve. The release curve was plotted based on the calculated QT content.

### Cellular Uptake and Cytotoxicity In Vitro

To confirm the presence of CD44 on A549 cells, the expression of CD44 was detected according to established methods [22]. C/Q@ZIF-8 and C/Q@ZIF-8-HA need to be labeled with FITC for imaging using a confocal microscope (FV1000, Olympus). Confocal microscopy was applied to observe the cellular uptake of targeted nanozymes. As well, the cytotoxicity of C/Q@ZIF-8 and C/Q@ZIF-8-HA in A549 and NHBE cells was assessed by the CCK-8 assay. All experiments were repeated three times.

### Cell colony formation assay and proliferation assay

Cell proliferation was detected with CCK-8 assay kit. A549 cells were inoculated into 96-well plates at a density of 3000 cells/well with 100 µl of culture medium. Cell viability of each group was measured every 24 h, and absorbance was measured at 450 nm.

To determine the clone-forming ability of cells in different treatment groups, cells were inoculated in 6-well plates. The culture medium was changed every 3 days and the cells were cultured for 14 days until the colonies were clearly visible. Cells were washed with PBS and then fixed using 4% paraformaldehyde, stained with crystal violet for 30 min, and colonies of > 50 cells were counted. All experiments were repeated three times.

### Cell apoptosis assay

Apoptosis was assessed using flow cytometry and Annexin V-FITC/PI Apoptosis Detection Kit. In brief, cells were collected and suspended in 200 µl of buffer including 5 µl of Annexin V-FITC and 5 µl of PI. Cells were then incubated for 15 min at room temperature and shielded from light. Cells were analyzed for apoptosis using FlowJo software.

### Western blotting assay

Cells were collected and lysed with RIPA buffer (Beyotime, China) containing protease inhibitors on ice for 30 min. Total protein was quantified according to the instructions of the Bradford kit. Protein samples were separated by 10% gel electrophoresis and transferred to PVDF membranes. Blotted membranes were closed in 5% BSA for 2 h and then incubated with primary antibody (1:1000) overnight at 4℃. The blotted membranes were then incubated with secondary antibody (1:5000) for 2 h at room temperature and the intensity of the bands was detected using a chemiluminescent substrate (Thermo Fisher, USA).

The primary antibodies used were against the following antigens: Cleaved PARP (Cell Signaling Technology, USA), γH2AX (Proteintech, USA), and GAPDH (Proteintech, USA).

### Establishment of Tumor models

A mouse subcutaneous tumor model was established by combining nanoparticles with X-ray irradiation, and 6-week-old female mice were randomly divided into 5 groups of 6 mice each. A549 cells (1 × 10^7^, 100 µL) were injected subcutaneously into the back of the mice to establish the A549 tumor-bearing mouse model. When the tumor volume of the mice reached approximately 50 mm^3^, 100 mL of nanoparticles were injected into the mice through intravenous administration at a dose of 0.5 mg/kg. Then, 6 Gy X-ray treatment was performed separately according to the grouping. To focus the radiation mainly on the tumors of the mice, the non-irradiated parts of the mice were shielded with lead plates. Tumor size was measured every 3 days, and no mice were excluded. All tumor tissues were fixed in 10% formalin, embedded in paraffin, and sectioned into continuous slices of 4 μm thickness. The paraffin sections were subjected to immunohistochemical (IHC) staining using standard methods. The pathologist was blinded to the identities and analyses of the pathology slides.

### In vivo biodistribution and Hematoxylin and Eosin (H&E) staining of organs

A549 tumor-bearing BALB/c mice were injected with C/Q@ZIF-8-HA nanoparticles. The fluorescence intensity of major organs and tumors in mice was measured using non-invasive near-infrared optical imaging system at 24 h, 72 h, and 15 days, respectively. N = 3 mice per group.

H&E staining were used to access the organ toxicity of RT and the sedimentation of C/Q@ZIF-8-HA in critical organs. On day 14, mice selected from each group were euthanized and their major organs (heart, liver, spleen, lung, and kidney) were harvested to test the toxicity of C/Q@ZIF-8-HA. To verify treatment efficacy, tumors from mice were also harvested for H&E staining. Formalin-fixed tissue sections were prepared and stained with hematoxylin and eosin. In the end, tumor cells were counterstained with nuclear fast red for 5 min.

### Statistical analysis

Statistical analysis was performed using SPSS 22.0. All data were presented as mean ± SD. Comparisons between two groups were performed using the Student’s *t*-test. Comparisons between three or more groups were performed using one-way analysis of variance (ANOVA). *P* < 0.05 indicates that the differences are statistically significant.

## Results and discussion

### Characterization and catalytic activity of C/Q@ZIF-8-HA

**Fig. 1:**
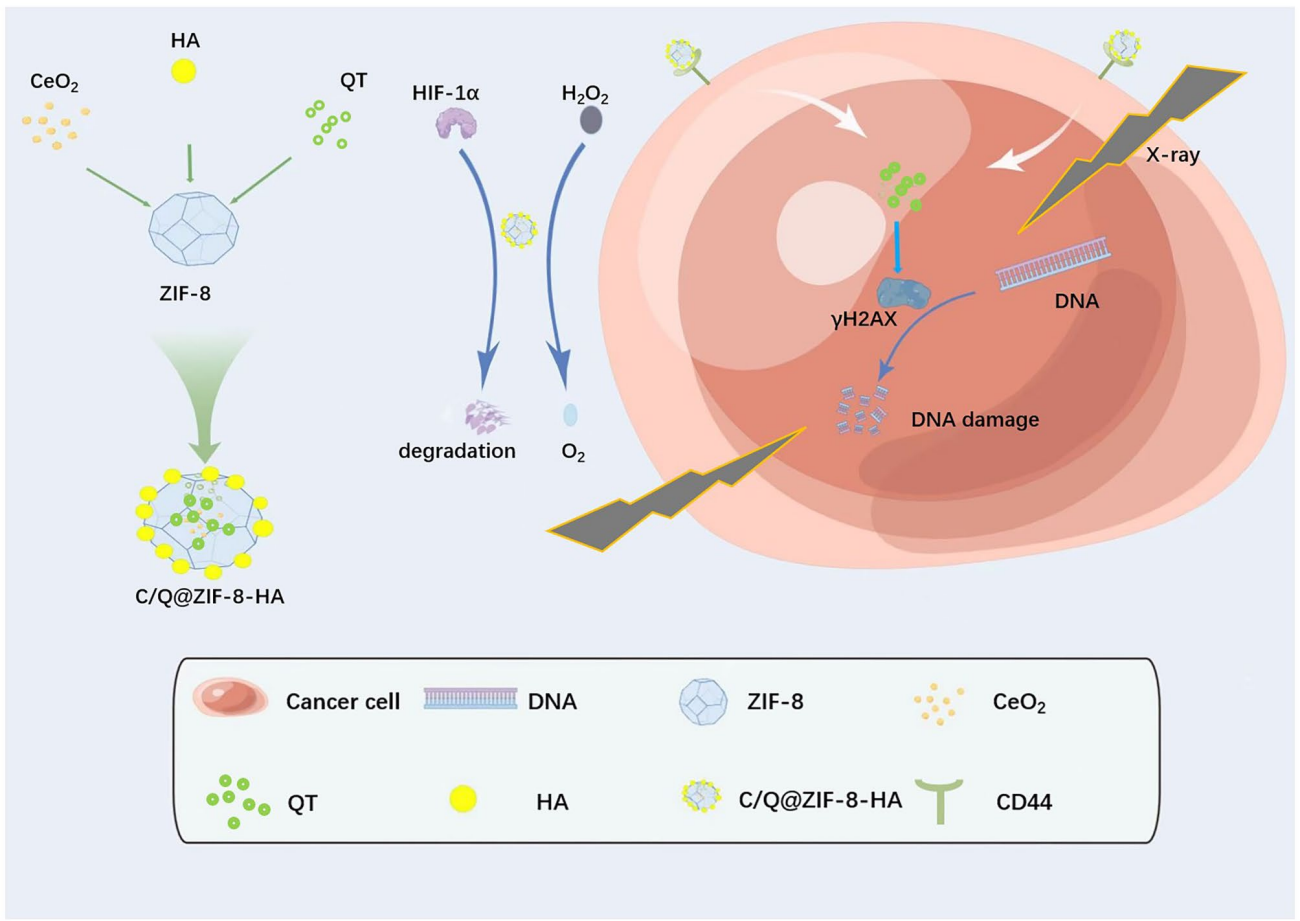
Schematic diagram of the sequential synthesis procedure of multifunctional radiosensitizers with peroxidase activity enhanced intracellular radiation deposition for efficient radiotherapy of NSCLC

**Fig. 2:**
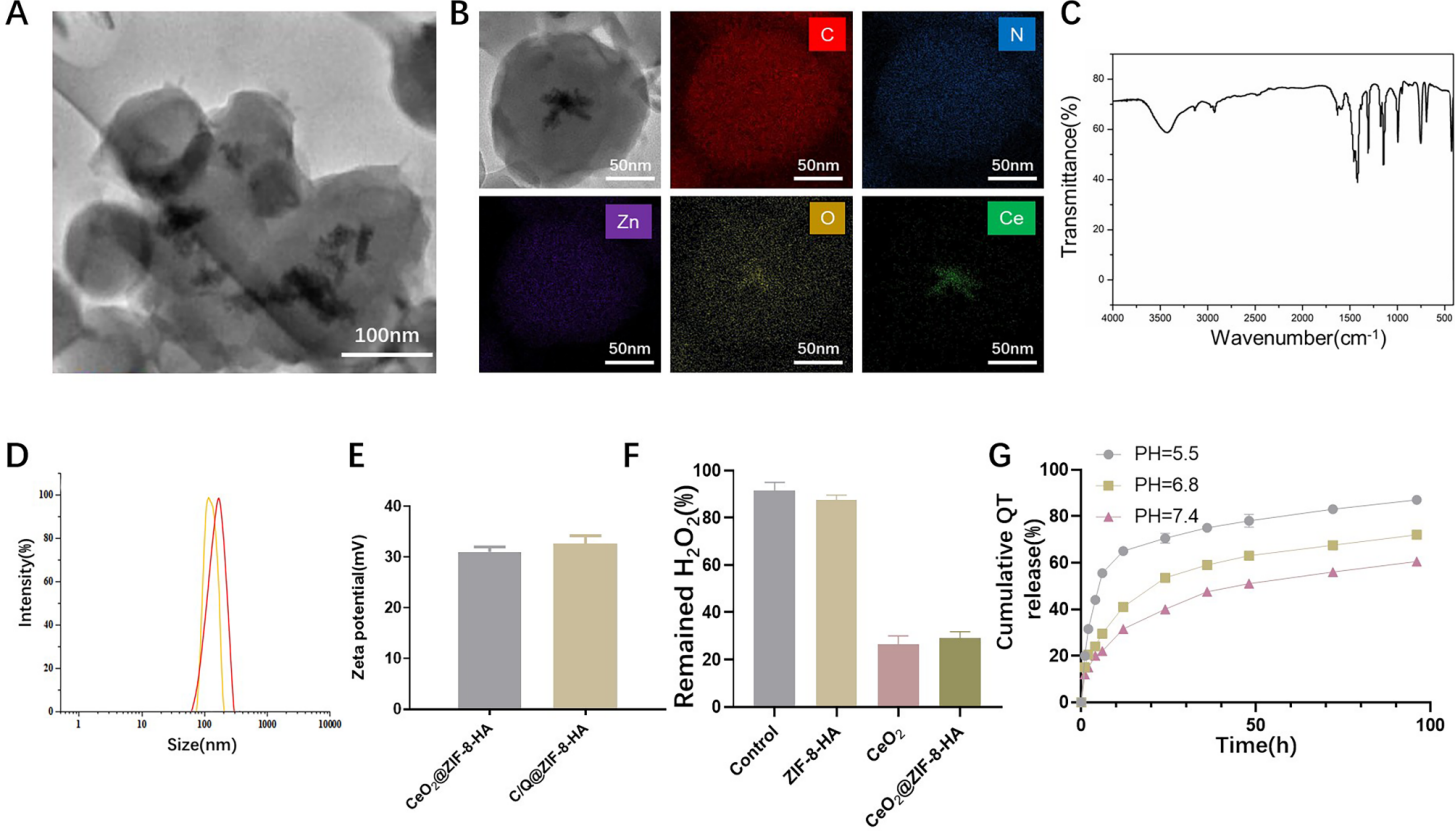
Characterization and catalytic activity of C/Q@ZIF-8-HA. (**A**) TEM image of C/Q@ZIF-8-HA. (**B**) TEM image and element mapping C/Q@ZIF-8-HA. (**C**) FT-IR of C/Q@ZIF-8-HA. (**D**) Particle size analysis of CeO_2_@ZIF-8-HA and C/Q@ZIF-8-HA. (**E**) Zeta potential analysis of CeO_2_@ZIF-8-HA and C/Q@ZIF-8-HA. (**F**) Catalase-mimicking activity of ZIF-8, CeO_2_, CeO_2_@ZIF-8-HA and C/Q@ZIF-8-HA. (**G**) In vitro release curve of QT. **P < 0.01

We have synthesized CeO_2_@ZIF-8 and C/Q@ZIF-8 nanoparticles using a “one-pot” method. Due to its good targeting and biocompatibility, HA has been widely used in tumor treatment. Therefore, we modified HA on the surface of the nanoparticles to fabricate CeO_2_@ZIF-8-HA and C/Q@ZIF-8-HA (Fig. 1). As shown in Fig. 2A, we characterized the morphology (size and shape) of C/Q@ZIF-8-HA and C/Q@ZIF-8-HA by TEM. Elemental mapping images show that C, N, O and Zn elements are uniformly distributed in C/Q@ZIF-8-HA nanoparticles, while Ce element is distributed inside the nanoparticles (Fig. 2B). The FT-IR spectra in Fig. 2C show the hydrogen skeleton vibration of the amine group of C/Q@ZIF-8-HA at 3327 cm^− 1^ and the vibration peak of the carbonyl group at 1420 cm^− 1^, further confirming the successful synthesis of C/Q@ZIF-8-HA. The average hydrodynamic particle diameter of was approximately 108 nm according to DLS measurements (Fig. 2D). After loading QT, the average hydrodynamic particle diameter of C/Q@ZIF-8-HA increased to approximately 132 nm. The zeta potentials of CeO_2_@ZIF-8-HA and C/Q@ZIF-8-HA were 31.0mV and 32.2mV, respectively, suggesting their excellent uniformity and stability (Fig. 2E). Due to the high porosity of ZIF-8, the CeO_2_ loading amount of C/Q@ZIF-8-HA was calculated as 17.7% and the QT loading amount as 11.2%. The above experimental results have confirmed the successful fabrication of C/Q@ZIF-8-HA nanoparticles.

As is known to all, Fenton-like reactions are a general term for reactions in which some transition metals such as Cu, Mn and Ce can accelerate or replace Fe(II) and catalyze the H_2_O_2_ reaction [23]. The level of H_2_O_2_ in solid tumors is generally higher than in normal tissues, and considering the increased level of H_2_O_2_ in TME, a Fenton-like response could be utilized to achieve highly selective sensitization by RT. Fenton-like catalysts show great promise for clinical applications. Among the catalysts reported so far, Fenton-like catalysts show great promise for clinical applications. Zheng et al. synthesized an MnO-based nanoparticle as an inducer of iron toxicity, which can specifically release Mn via depletion of glutathione and then activate Fenton-like reactions in the TME [24]. We tested the catalytic ability of C/Q@ZIF-8-HA nanoparticles in vitro using 100 mmol/L H_2_O_2_ to simulate the H_2_O_2_ content in TME. The catalytic activity of the reaction solution containing CeO_2_@ZIF-8-HA and C/Q@ZIF-8-HA towards H_2_O_2_ was significantly higher compared to the other nanoparticles. As shown in Fig. 2F, the decomposition rate of H_2_O_2_ for C/Q@ZIF-8-HA is significantly higher than that of the control group, but slightly lower than that of CeO_2_ group. This indicates that the CeO_2_ dispersed in ZIF-8 does not have a significant impact on its catalytic ability. Consequently, these results indicate that the C/Q@ZIF-8-HA nanozyme exhibited superior nanozyme activity in decomposing H_2_O_2_ to O_2_. Next, we measured the release of QT from C/Q@ZIF-8-HA nanoparticles under different pH conditions. As shown in Fig. 2G, the release amounts reached 87.1% and 72.7% at pH 5.5 and pH 6.8, respectively, after 96 h, while the cumulative release amount at pH 7.2 was 60.5%, indicating that acidic conditions were more favorable for the release of QT. This indicates C/Q@ZIF-8-HA nanoparticles have the function of pH-responsive release, allowing targeted release of the encapsulated QT.

### In Vitro tumor cells internalization

**Fig. 3:**
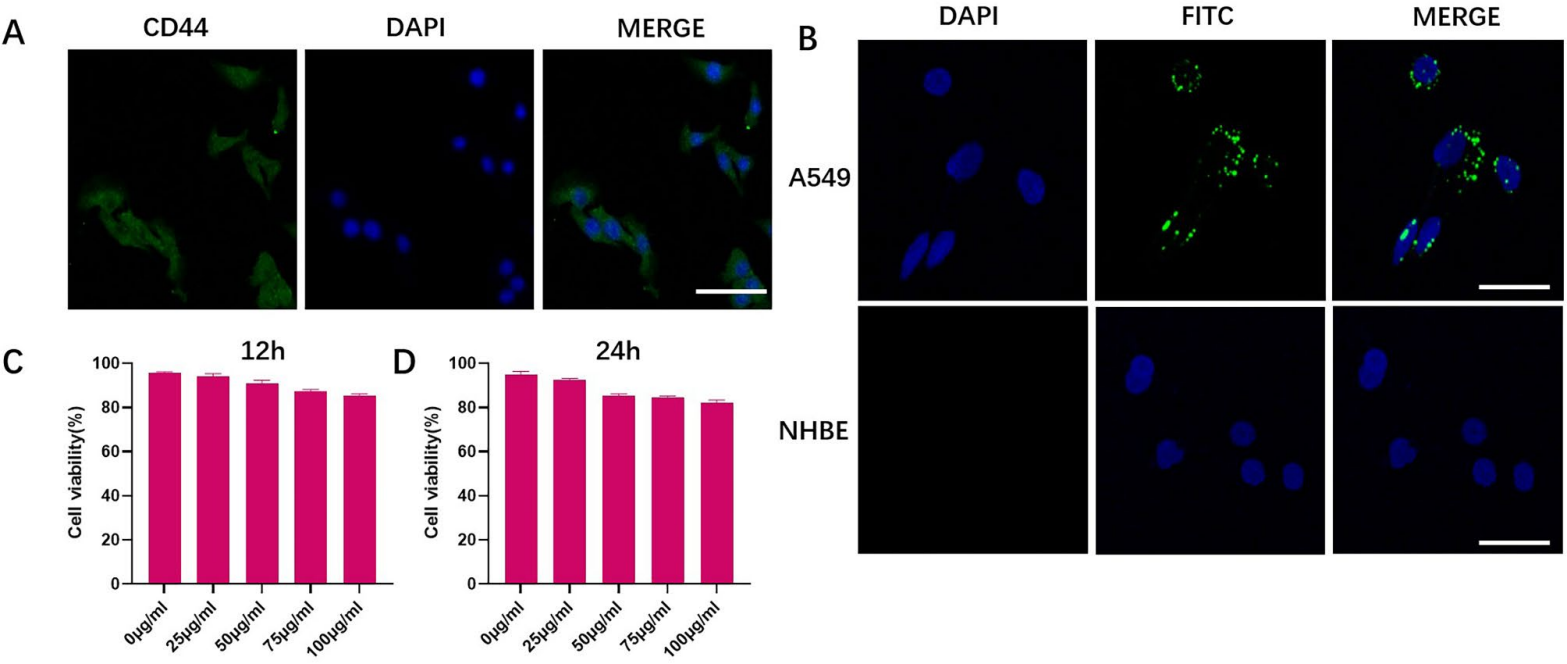
(**A**) Immunofluorescence microscopy image of A549 cells stained by CD44. (**B**) Immunofluorescence images of cultured A549 cells after 4 h of C/Q@ZIF-8-HA treatment. (**C**) and (**D**) The biocompatibility of C/Q@ZIF-8-HA on A549 cells at different concentration (0, 12.5, 25, 50, 75 and 100 µg ml^− 1^) after incubation for 12 and 24 h. Scale bar: 20 μm

As the passive targeting strategy has limited internalization of tumor cells due to the EPR effect of the solid tumor [25], we constructed an active targeting strategy that relies on a receptor-ligand mechanism, using HA with a molecular weight of approximately 200 kDa to modify the C/Q@ZIF-8. CD44 is a complex transmembrane adhesion glycoprotein expressed in a variety of human cells, including embryonic stem cells, differentiated cells and cancer cells. As recognized marker of tumor stem cells, CD44 is a critical regulator of epithelial mesenchymal transition (EMT), involved in tumorigenesis, progression, and metastasis. Zeng et al. constructed a nanoplatform consisting of copper-doped mesoporous Prussian blue (CMPB) encapsulated glucose oxidase (GOx) and a HA coating modified with a nitric oxide donor. The HA coating enables this nanoparticles to actively target CD44 receptors in cancer cells and also enhances vascular permeability [26]. Firstly, immunofluorescence assay was conducted by us to detect the expression of CD44 on the surface of A549 cells. As shown in Fig. 3A, the surface of A549 cells stained intensely, indicating adequate CD44 expression. The tumor cell targeting ability of nanoparticles was investigated with FITC-labeled C/Q@ZIF-8-HA. Normal human bronchial epithelial cell (NHBE) was used to verify whether the enhanced uptake of C/Q@ZIF-8-HA was restricted to tumor cells, but not normal lung epithelial cells. As illustrated in Fig. 3B, FITC-labeled C/Q@ZIF-8-HA-treated A549 cells showed a strong green fluorescent signal in the cytoplasm. However, NHBE cells treated with C/Q@ZIF-8-HA had no FITC fluorescence signal. These phenomena suggest that C/Q@ZIF-8-HA could significantly increase the uptake of C/Q@ZIF-8 by A549 cells through the targeted binding of HA and CD44. Furthermore, the increased uptake of C/Q@ZIF-8 was restricted to tumor cells, but not to normal lung epithelial cells. Next, we evaluated the cytotoxicity profile of C/Q@ZIF-8-HA by CCK-8 assay, which is an important parameter for nano-radiosensitizers towards clinical applications and to improve the effectiveness of RT on tumor cells. Cell survival was examined after incubation of A549 with C/Q@ZIF-8-HA for 12 or 24 h. The cell survival rate reached more than 83% at all concentrations of C/Q@ZIF-8-HA from 0 to 100 µg/mL, indicating that C/Q@ZIF-8-HA has good cytocompatibility even at comparatively higher concentrations (Fig. 3C and D).

### C/Q@ZIF-8-HA nanozymes attenuate hypoxia and enhance radiosensitivity in vitro

**Fig. 4:**
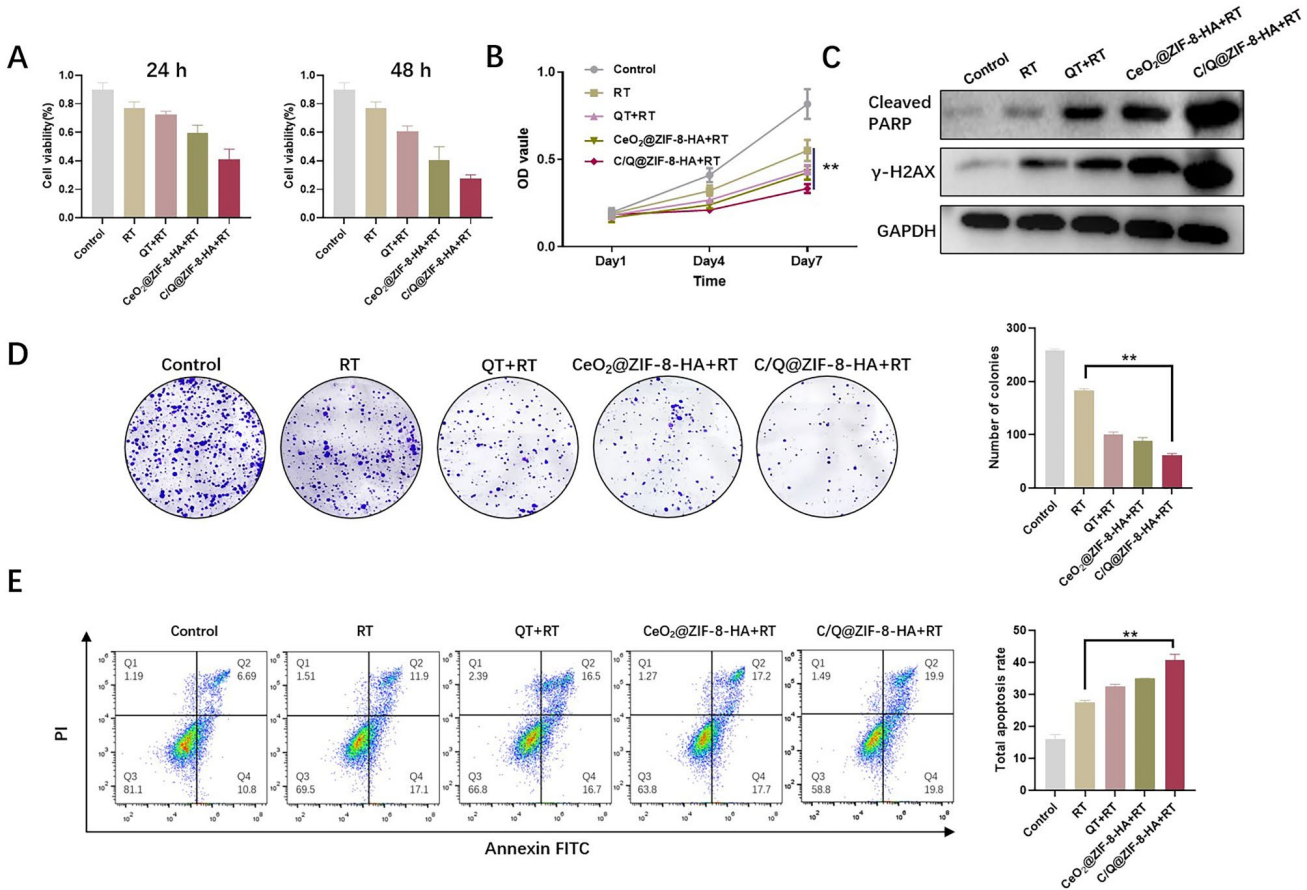
(**A**) Representative results of the A549 MTT assay in different groups including the control, RT, QT + RT, CeO_2_@ZIF-8-HA + RT, and C/Q@ZIF-8-HA + RT groups. The results from 24 and 48 h time points showing statistically significant difference. (**B**) CCK-8 assay was used to analyze the proliferation ability of five groups. (**C**) Western blotting analysis of cleaved PARP and γH2AX in control, RT, QT + RT, CeO_2_@ZIF-8-HA + RT, and C/Q@ZIF-8-HA + RT groups, respectively. (**D**) A colony of A549 cells treated with QT, CeO_2_@ZIF-8-HA, or C/Q@ZIF-8-HA combined X-ray (6 Gy). (**E**) Annexin V-FITC/PI staining was performed to determine the apoptosis of A549 cells in control, RT, QT + RT, CeO_2_@ZIF-8-HA + RT, and C/Q@ZIF-8-HA + RT groups. **P < 0.01

In the in vitro experiments we have set up a total of five subgroups: Group (a): wild type A549 cells; Group (b): a + X-ray(6 Gy); Group (c): b + QT; Group (d): b + CeO_2_@ZIF-8-HA; Group (e): b + C/Q@ZIF-8-HA. For investigating the sensitizing effect of C/Q@ZIF-8-HA on NSCLC radiotherapy, we used in vitro cellular assays. QT, a flavonoid aldose reductase inhibitor of natural plant origin, has a variety of antitumor activities, presumably due to its ability to inhibit the MAPK and Akt signaling system activity in a variety of tumor cells [27]. To verify the synergistic sensitizing effect of QT and CeO_2_ on RT in vitro, we co-cultured C/Q@ZIF-8-HA with A549 cells for 24 and 48 h. At these two time points, the cell survival rate of C/Q@ZIF-8-HA + RT group was significantly lower than that of RT alone. Accordingly, the cell survival rate in the C/Q@ZIF-8-HA + RT group was also significantly lower than the cell survival rate in CeO_2_@ZIF-8-HA + RT and QT + RT groups (Fig. 4A). These results tentatively indicate that combined RT with C/Q@ZIF-8-HA could exert the combined sensitizing effect of QT and CeO_2_ to maximize tumor cell killing and inhibit cell proliferation. We next sought to investigate the effects of C/Q@ZIF-8-HA on the proliferation of A549 cells. C/Q@ZIF-8-HA significantly inhibited the rate of cell proliferation compared with the control group. Accordingly, The CeO_2_@ZIF-8-HA + RT and QT + RT groups showed higher inhibition of cell proliferation than the RT group, but weaker than the C/Q@ZIF-8-HA group (Fig. 4B).

Targeted DNA repair pathways have shown great potential in enhancing the effects of radiation therapy on tumor cells. Poly ADP-ribosepolymerase (PARP) is a multifunctional nuclear protein that plays an essential role in DNA damage repair. Ionizing radiation can cause intracellular DNA double-strand breaks to exert its therapeutic effect, and DNA repair after damage can affect the efficacy of radiotherapy. γ-H2AX is the most effective early biomarker for detecting DNA double-strand breaks and is associated with tumorigenesis, progression and apoptosis [7]. We employed Western Blot assay to evaluate the impact of C/Q@ZIF-8-HA on the expression of Cleaved PARP and γ-H2AX. As shown in Fig. 4C, Cleaved PARP and γ-H2AX were significantly elevated in C/Q@ZIF-8-HA + RT group, indicating that C/Q@ZIF-8-HA significantly enhanced DNA double-strand breaks in tumor cells induced by ionizing radiation.

Subsequently, we evaluated the ability of C/Q@ZIF-8-HA to enhance the radiosensitivity of A549 cells through clonogenic assay. In the control group, the number of A549 cell colonies was approximately 275, while in the RT group, it was approximately 164. In contrast, the number of colonies of the C/Q@ZIF-8-HA + RT group was 64, which was significantly lower than that of the QT + RT (92) and CeO_2_@ZIF-8 + RT groups (87) (Fig. 4D). Figure 4E shows that the apoptosis rates of different groups of cells were compared by flow cytometry experiments. Similar to the CCK-8 assay results, the apoptosis rate of A549 cells reached 31.2% and 33.4% after QT + RT or CeO_2_@ZIF-8 + RT treatment, respectively, which was significantly different compared to the RT group. However, a significant increase in the total apoptosis rate (39.7%) was observed in the C/Q@ZIF-8-HA + RT group, which was almost 1.5-fold higher than that in the RT group and 2.5-fold higher than that in the control group.

These results suggest that CeO_2_ and QT have potential as radiosensitizers. ZIF-8 encapsulated with CeO_2_ and QT can better exert the synergistic effect of both radiotherapy sensitizers. On the one hand, CeO_2_ can improve tumor hypoxia, increase hydroxyl radical production and increase DNA uptake for ionizing radiation capacity, and on the other hand, QT can maximally induce tumor cell apoptosis by increasing X-ray uptake, thus achieving the purpose of radiation sensitization.

### C/Q@ZIF-8-HA attenuated TME hypoxia and enhanced therapeutic efficacy in vivo

**Fig. 5:**
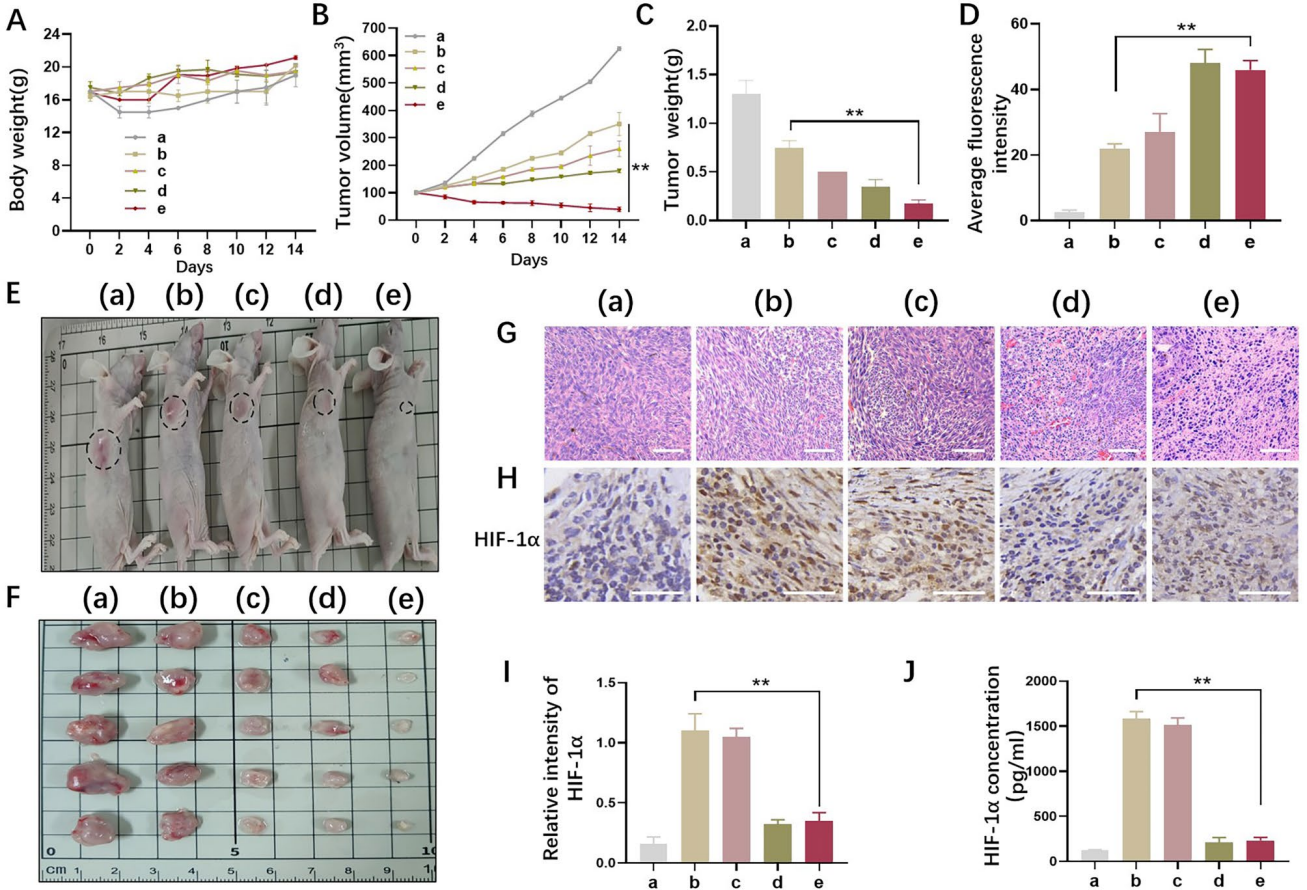
A549 tumor-bearing BALB/c nude mice were injected intravenously with C/Q@ZIF-8-HA. The tumor-bearing BALB/c nude mice were randomly divided into five groups: (a) control group, (b) RT alone group, (c) QT + RT group, (d) CeO_2_@ZIF-8-HA + RT group, and (e) C/Q@ZIF-8-HA + RT group. (**A**) Body weight of A549 tumor-bearing BALB/c nude mice in each group over 14 days after treatment. (**B**) Tumor weights of different groups in A549 tumor-bearing BALB/c nude mice. (**C**) Tumor volumes of different groups in A549 tumor-bearing BALB/c nude mice. (**D**) ROS levels in tumor tissue after treatment in different groups. (**E-F**) Tumors were collected from five different groups 14 days after different treatments. The images of the tumors are displayed. (**G**) Representative hematoxylin-eosin staining in NSCLC tissue in five groups. Scale bar: 100 μm. (**H-I**) Representative HIF-1α immunohistochemical staining and relative expression intensity were observed in the 5 groups of tumor tissues. Scale bar: 20 μm. (**J**) The enzyme-linked immunosorbent assay of HIF-1α in five groups in tumor tissue. **P < 0.01

Inspired by the effectiveness of C/Q@ZIF-8-HA combined with RT from our in vitro results, the therapeutic potential of dual sensitization of C/Q@ZIF-8-HA in vivo was further investigated by intravenous administration. The tumor-bearing BALB/c nude mice were randomly divided into five groups: (a) control group, (b) RT alone group, (c) QT + RT group, (d) CeO_2_@ZIF-8-HA + RT group, and (e) C/Q@ZIF-8-HA + RT group. As shown in Fig. 5A, there was no statistically significant difference in body weight of mice among the five groups after 14 days of treatment. Not only that, sensitized nanoparticles did not affect the survival of mice in each group. Notably, as shown in, the mean tumor volume and tumor weight in the C/Q@ZIF-8-HA group were the lowest among all five groups (Fig. 5B C), suggesting that the synergistic effect of the C/Q@ZIF-8-HA dual sensitizing nanoparticles enhanced the efficacy of RT and provided an advantage in tumor eradication.

Radiation generates a significant amount of ROS in RT, which can directly or indirectly cause DNA damage and cell apoptosis, thereby disrupting the growth and metastasis of cancer cells. Additionally, ROS can induce a cascade of signal pathways and inflammatory reactions, which further enhance the therapeutic effect. Therefore, ROS plays a critical role in RT. Using flow cytometry, ROS levels were measured in various tumor tissues. The results showed that the relative fluorescence intensities in the control group, RT group, and QT + RT group were 2.1 ± 1, 20.3 ± 1.7, and 23.1 ± 3.6, respectively. The ROS content in the CeO_2_@ZIF-8-HA + RT group and C/Q@ZIF-8-HA + RT group was significantly higher than that of the RT group (P < 0.01) (Fig. 5D).

Compared with the control group, the tumor growth inhibition values were 27.5% and 36.8% in the QT + RT and CeO_2_@ZIF-8-HA + RT groups, respectively. And more significantly, the tumor growth inhibition value of C/Q@ZIF-8-HA + RT group reached 77.3%, compared to the RT group. Accordingly, the general tumor specimens also showed such changes, as shown in Fig. 5E and F. The sensitizing effect of radiotherapy was most noticeable in the C/Q@ZIF-8-HA group. Next, hematoxylin-eosin (H&E) staining was used to assess the radiosensitizing effect of C/Q@ZIF-8-HA (Fig. 5G). Tumor necrosis and inflammatory cell infiltration were significantly increased in the RT group, QT group and CeO_2_@ZIF-8-HA group compared to the control group. More significant increased necrosis, cellular alterations, and nuclear condensation were observed in the C/Q@ZIF-8-HA group. These results suggest that this dual sensitization strategy can improve the effectiveness of radiation therapy for tumors.

Hypoxia-inducible factor-1α (HIF-1α) is a key transcriptional regulator that mediates the cellular hypoxic response. Under normoxic conditions HIF-1α is prone to enzymatic degradation; but under hypoxic conditions, HIF-1α protein remains highly active in tumors. Improving TME hypoxia is the key to achieving tumor radiosensitization. To detect the oxygenating ability of C/Q@ZIF-8-HA in vivo, we examined the expression of HIF-1α by immunohistochemistry assay and ELISA assay. As shown in Fig. 5H and I, HIF-1α expression was low in group a (wild-type A549) and significantly increased in A549 cells after X-ray irradiation, suggesting that hypoxia is a typical feature of tumor cell resistance to RT and a critical factor in radiosensitization. Moreover, the expression of HIF-1α in X-ray irradiated A549 cells was significantly reduced after treatment with C/Q@ZIF-8-HA. We further obtained results similar to immunohistochemistry assay by detecting the expression of HIF-1α in tumors through ELISA assay (Fig. 5J).

**Fig. 6:**
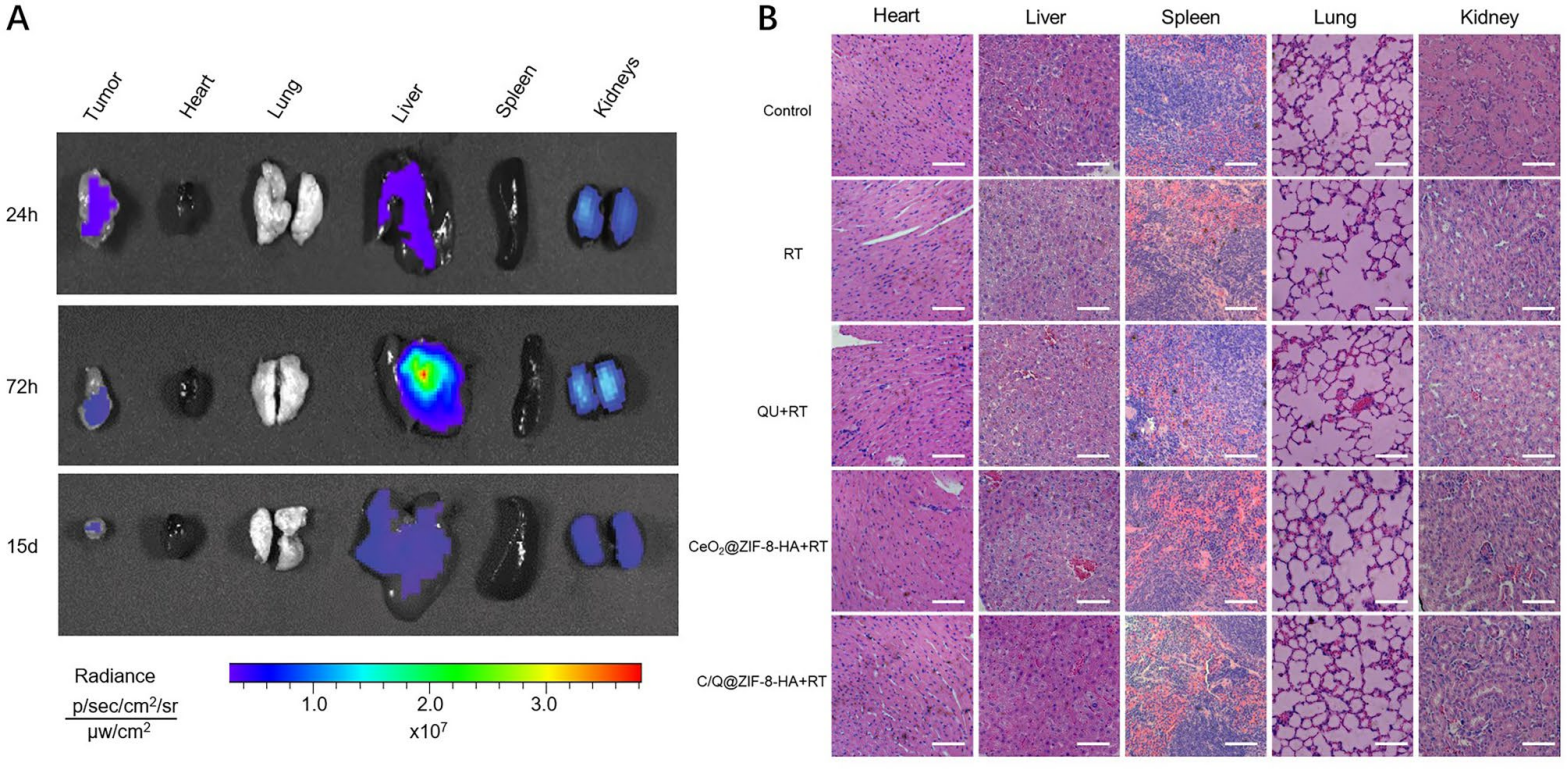
(**A**) NIR fluorescence images of tumors and five different organs. (**B**) H&E staining of various treatment groups. Scale bar: 100 μm

Evaluating the safety of nanoparticles in biomedical applications is essential. To investigate the biodistribution of nanoparticles, C/Q@ZIF-8-HA was injected into tumor-bearing mice. Animals were sacrificed at designated time points after injection, and the fluorescence intensity of Cy5 in isolated tissues was measured. As shown in Fig. 6A, the fluorescence intensity was weak in the heart, lungs, and spleen, while relatively high in the tumor, liver, and kidneys. Next, we utilized H&E staining to assess the potential organ toxicity and deposition of nanoparticles in critical organs. Mice were sacrificed 14 days after nanoparticles injection, and heart, liver, spleen, lung and kidney tissues were taken for histological observation and analysis. As shown in Fig. 6B, histopathological analysis of the major organs showed that C/Q@ZIF-8-HA injection did not result in significant pathological changes compared to other treatment groups, indicating its favorable biocompatibility.

In the histopathological evaluation, none of the four treatment groups caused pathological changes compared to the control group. Compared to more studied tumors, such as cervical, prostate and nasopharyngeal cancers, NSCLC is inherently difficult to investigate because it is generally located deep in the body cavity, rather than on the surface, and local injection is difficult to achieve. Hence, our study evaluated the targeting and safety of intravenous administration of C/Q@ZIF-8-HA, and the results suggest its potential for intravenous administration in NSCLC. Finally, C/Q@ZIF-8-HA not only improves local tumor control, but also reduces the dose of RT without compromising the tumor suppression effect. Reducing the radiation dose could lead to lower toxicity and better immune system protection, thus increasing the potential for antitumor immunity.

## Conclusions

In conclusion, this study developed a novel radiosensitization approach that combines CeO_2_ loaded ZIF-8 nanozymes and QT to form a core-shell structure with anti-hypoxia and enhanced DNA damage properties, resulting in effective radiosensitization for radiotherapy. ZIF-8 offers the basic core-shell structure that facilitates the duration of the nanoparticles in the tumor. The modification of HA ensures optimal targeting of the nanoparticles to A549 cells. These C/Q@ZIF-8-HA nanozymes, while efficiently targeting tumor cells, significantly decrease HIF-1α by reducing intracellular and TME hypoxia, thus enhancing X-ray killing of tumor cells. In conclusion, C/Q @ZIF-8-HA, which has the therapeutic advantage of nanozymes combined with QT, significantly promotes DNA damage and overcomes hypoxia-induced radioresistance in vitro and in vivo, and synergistically improves therapeutic efficacy of radiotherapy for NSCLC tumors. This multifunctional nanozyme provides a new approach for radiotherapy sensitization of NSCLC in future clinical treatment.

## Data Availability

All data generated or analyzed during this study are included in this published article.
